# Case Report: Prolonged survival following multimodal treatment of leptomeningeal metastases in triple-negative glycogen-rich clear cell breast carcinoma

**DOI:** 10.3389/fonc.2026.1901536

**Published:** 2026-10-22

**Authors:** Yun-Chih Lo, Chun-Chieh Wu, Shang-Chun Hung, Lu-Pei Pan, Shen-Liang Shih

**Affiliations:** 1 Division of Breast Oncology and Surgery, Department of Surgery, Kaohsiung Medical University Chung-Ho Memorial Hospital, Kaohsiung, Taiwan; 2 Department of Pathology, Kaohsiung Medical University Chung-Ho Memorial Hospital, Kaohsiung, Taiwan; 3 Department of Medical Imaging, Kaohsiung Medical University Chung-Ho Memorial Hospital, Kaohsiung, Taiwan; 4 Division of Breast Oncology and Surgery, Department of Surgery, Kaohsiung Medical University Gangshan Hospital, Kaohsiung, Taiwan

**Keywords:** case report, glycogen-rich clear cell carcinoma, leptomeningeal metastases, sacituzumab govitecan, triple-negative breast cancer

## Abstract

Leptomeningeal metastases (LM) are a devastating complication of advanced breast cancer and confer an especially poor prognosis in patients with triple-negative breast cancer (TNBC). Therapeutic options for LM remain limited. Glycogen-rich clear cell (GRCC) carcinoma of the breast is an exceptionally rare histological subtype, with poorly defined metastatic patterns and optimal management strategies. This report presents a case of TNBC exhibiting GRCC characteristics, further complicated by LM. A 51-year-old woman was diagnosed with stage IV TNBC with GRCC morphology and concomitant axillary lymph node and liver metastases. Ten months after the initial diagnosis, LM was confirmed by brain magnetic resonance imaging and cerebrospinal fluid cytology. Marked clinical improvement was achieved following ventriculoperitoneal (VP) shunt placement and systemic treatment with sacituzumab govitecan (SG). The patient survived for 21 months since the diagnosis of LM and maintaining good functional status until the final month of her life. To our knowledge, this is the first reported case of LM arising from GRCC carcinoma of the breast. This case suggests a potential therapeutic role for SG in LM associated with TNBC and raises the hypothesis that VP shunting may enhance treatment efficacy by altering drug exposure. Further studies are warranted to validate these findings.

## 1 Introduction

Glycogen-rich clear cell (GRCC) carcinoma is an uncommon histological subtype of breast cancer, characterized by tumor cells containing abundant intracytoplasmic glycogen that results in a clear cell appearance. According to the World Health Organization classification, the diagnosis of GRCC requires that more than 90% of tumor cells demonstrate this distinctive morphology (1). GRCC accounts for approximately 1.4–3% of all breast malignancies and remains poorly characterized, with most existing knowledge derived from isolated case reports and small retrospective series (2). Consequently, its biological behavior, prognostic implications, and metastatic patterns remain incompletely understood and somewhat controversial. Leptomeningeal metastases (LM), particularly in the absence of parenchymal brain metastases, represent a rare but clinically devastating manifestation of advanced breast cancer and are more frequently observed in triple-negative subtypes (3). Current treatment options include systemic therapy, intrathecal chemotherapy and radiotherapy. To date, there is a paucity of data regarding the occurrence and clinical course of LM in patients with GRCC carcinoma of the breast.

Sacituzumab govitecan (SG), a Trop-2–directed antibody–drug conjugate (ADC), has demonstrated significant clinical benefit in patients with metastatic triple-negative breast cancer (TNBC) as a second-line or later treatment (4). The standard dosage is 10 mg/kg intravenously on days 1 and 8 of each 21-day cycle. However, its efficacy in the treatment of LM remains largely unexplored, because patients with leptomeningeal involvement have been routinely excluded from prospective trials. Herein, we report a patient with metastatic TNBC exhibiting GRCC features, initially involving distant lymph nodes and the liver, who subsequently developed extensive LM ten months after diagnosis. This case underscores the potential for aggressive leptomeningeal dissemination in GRCC carcinoma of the breast and provides valuable clinical insight into the possible therapeutic role of SG in this challenging clinical setting.

## 2 Case description

A 51-year-old woman with no significant past medical history presented with a palpable painful mass measuring approximately 2 cm in diameter in the upper outer quadrant of the right breast. On physical examination, no overlying skin changes or nipple discharge were observed. Mammography revealed a 2.5cm-oval, equal-density mass with obscured margin in the upper outer quadrant of the right breast. Breast ultrasonography revealed a highly suspicious lesion, categorized as American College of Radiology Breast Imaging Reporting and Data System (ACR BI-RADS) category 4c. Core needle biopsy demonstrated a histological grade 2 invasive carcinoma with GRCC features (**Figure 1**). Immunohistochemical analysis showed a triple-negative phenotype: estrogen receptor expression was 0%, progesterone receptor expression was 0%, and the human epidermal growth factor receptor 2 (HER2) immunohistochemistry score was 0. The Ki-67 proliferation index was approximately 70%. Programmed death-ligand 1 (PD-L1) expression was negative, with a tumor proportion score (TPS) of <1% and a combined positive score (CPS) of <1. She was premenopausal at diagnosis, with menarche at 14 years of age. Her reproductive history was gravida 1, para 1, and her first full-term pregnancy at 32 years of age. Her family history was significant for lung cancer and colorectal cancer, with no known family history of breast or ovarian cancer.

**Figure 1:**
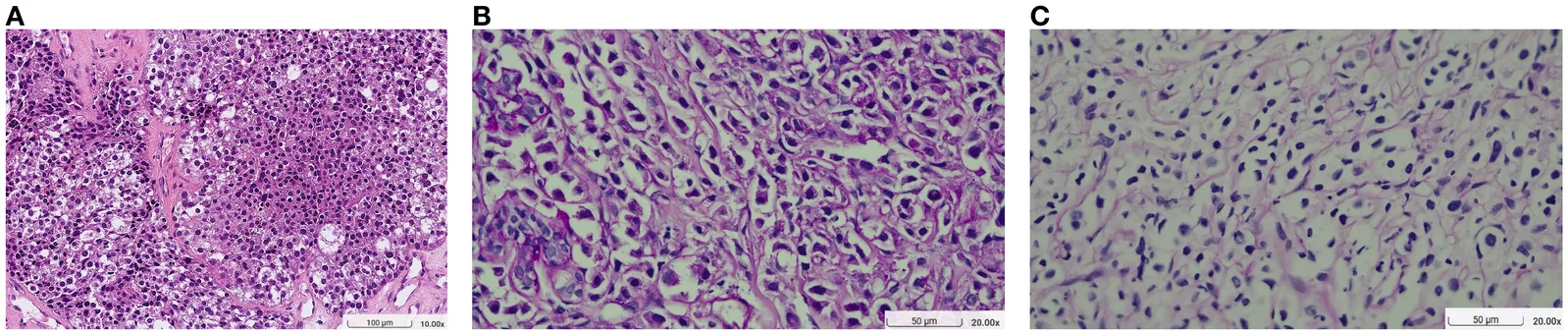
Histopathologic features of the breast tumor. **(A)** The tumor is composed of pleomorphic carcinoma cells with abundant clear cytoplasm arranged in solid sheets and tubular patterns (Hematoxylin and eosin stain; original magnification, 10x objective). **(B)** The glycogen is demonstrated by PAS stain in the clear cytoplasm (Periodic Acid-Schiff stain; original magnification, 20x objective). **(C)** The glycogen in the cytoplasm is diastase-sensitive. (Periodic acid Schiff plus diastase stain; original magnification, 20x objective).

Contrast-enhanced computed tomography (CT) revealed multifocal tumors in the right breast, the largest measuring 2.4 cm, accompanied by metastatic involvement of level I, II, and III axillary lymph nodes as well as right posterior cervical lymph nodes. Because hepatic nodules detected on CT and subsequent abdominal ultrasonography were suspicious for metastases, dynamic contrast-enhanced abdominal magnetic resonance imaging (MRI) was performed for further characterization. MRI demonstrated a small enhancing lesion in segment VII of the liver, consistent with metastatic disease (**Figure 2A**). Subsequent ^18^F-FDG PET/CT showed no discernible FDG uptake in the hepatic lesion. However, given the small size of the lesion, the absence of FDG avidity did not exclude metastatic disease. No liver biopsy was performed; therefore, the diagnosis remained radiological. Brain MRI obtained during initial staging showed no evidence of intracranial metastasis (**Figure 3A**). Germline genetic testing did not identify any pathogenic variants in BRCA1 or BRCA2. Based on these findings, the patient was diagnosed with stage IV TNBC (cT2N3M1) with GRCC features.

**Figure 2:**
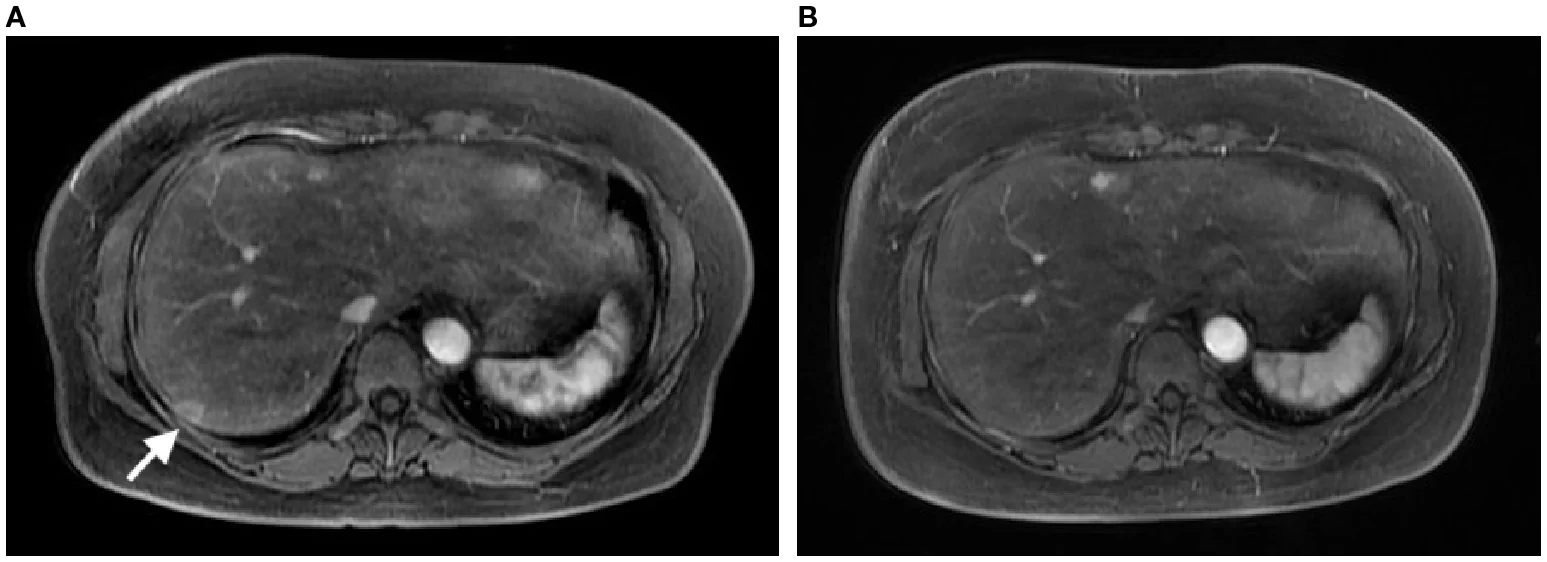
Serial dynamic contrast-enhanced abdominal MRI. Axial post-contrast T1-weighted fat-suppressed images obtained during the arterial phase show **(A)** A small enhancing nodule in hepatic segment VII that was radiologically suspicious for metastasis at diagnosis (white arrow). **(B)** Resolution of the metastatic lesion during SG therapy, with a persistent hematoma in segment IVa.

**Figure 3:**
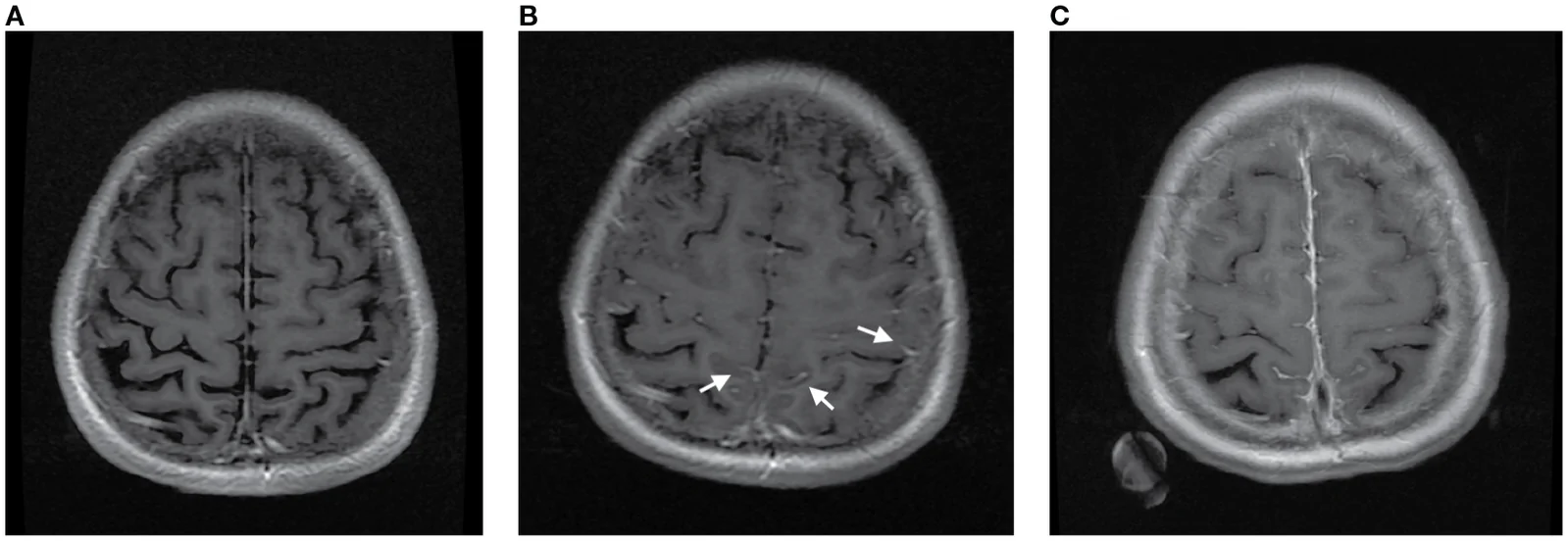
Serial contrast-enhanced brain MRI showing the evolution of LM. Axial post-contrast T1-weighted FLAIR images show **(A)** No evidence of brain metastasis at initial diagnosis. **(B)** No definite leptomeningeal enhancement was identified at the time of imaging, but subtle enhancement was suspected on retrospective review (white arrows). **(C)** Progressive pachymeningeal and leptomeningeal enhancement on follow-up MRI during SG treatment.

The patient initially received six cycles of triweekly systemic therapy consisting of docetaxel 75 mg/m^2^, carboplatin AUC 5, and bevacizumab 7.5mg/kg. Follow-up chest CT showed a partial response (PR) according to the Revised Response Evaluation Criteria in Solid Tumors (RECIST) version 1.1, with reductions in the sizes of the right breast tumors and involved lymph nodes. She subsequently underwent right total mastectomy with axillary lymph node dissection, followed by deep inferior epigastric artery perforator (DIEP) flap reconstruction and prophylactic lymphovenous anastomosis (LVA) to reduce the risk of postoperative upper limb lymphedema. Histopathological examination of the surgical specimen confirmed GRCC morphology and a triple-negative immunophenotype, with a Ki-67 index of approximately 40%. Despite the radiographic tumor shrinkage, multiple foci of residual invasive carcinoma remained, with the residual invasive disease measuring 5.2 cm in greatest dimension. All 13 dissected axillary lymph nodes contained metastatic carcinoma with extranodal extension, indicating a substantial residual breast and nodal disease burden after initial systemic therapy. Postoperatively, the patient began a planned six-cycle regimen of triweekly liposomal doxorubicin 37.5 mg/m^2^ plus cyclophosphamide 600 mg/m^2^. Radiotherapy was initiated during systemic treatment and delivered concurrently over 30 fractions, with 50 Gy administered to the right supraclavicular nodal region and a simultaneous integrated boost to a total dose of 60 Gy administered to the tumor bed and residual axillary disease.

Approximately ten months after the initial diagnosis, following completion of radiotherapy but during the planned course of systemic therapy, the patient developed progressive headache, dizziness, left hand numbness, and a transient episode of dysarthria. Neurological examination revealed no focal deficits. Contrast-enhanced brain MRI was initially interpreted as showing no definite leptomeningeal enhancement. However, retrospective review suggested subtle meningeal enhancement (**Figure 3B**). Cerebrospinal fluid cytology revealed malignant cells, confirming the diagnosis of LM. Consequently, the planned liposomal doxorubicin and cyclophosphamide regimen was discontinued after two cycles, and intravenous SG was selected as the next systemic therapy.

However, prior to SG initiation, the patient experienced rapid neurological deterioration, including severe headache, intractable vomiting, diplopia, and psychotic manifestations such as visual and auditory hallucinations. Repeat brain MRI revealed progression of leptomeningeal disease accompanied by worsening hydrocephalus. In the setting of suspected increased intracranial pressure, corticosteroids and osmotic therapy were administered, followed by placement of a VP shunt for cerebrospinal fluid diversion. This intervention resulted in marked symptomatic improvement.

After neurological stabilization, systemic treatment with SG was initiated at 10 mg/kg intravenously on days 1 and 8 of each 21-day cycle. Following the third cycle of therapy, MRI demonstrated a PR according to RECIST version 1.1, characterized by a reduction in the size of the metastatic lesion in the right hepatic lobe, without evidence of peritoneal dissemination (**Figure 2B**). During subsequent follow-up, serial brain MRI revealed gradual radiographic progression of both pachymeningeal and leptomeningeal enhancement (**Figure 3C**). In response, intrathecal chemotherapy with methotrexate (MTX) was initiated. The patient declined whole-brain radiotherapy.

Despite radiographic progression of LM, the patient remained clinically stable with an Eastern Cooperative Oncology Group (ECOG) performance status 1 for the first 20 months following LM diagnosis. During this period, she experienced no recurrent headaches or new neurological deficits, and remained independent in activities of daily living. She received a total of eleven cycles of SG, two sessions of intrathecal MTX 12mg, and six cycles of trastuzumab deruxtecan (T-DXd) 5.4mg/kg. T-DXd was subsequently discontinued because of fatigue and financial considerations. She was then treated for two months with a metronomic oral VEX regimen comprising cyclophosphamide 50 mg once daily, capecitabine 500 mg three times daily, and vinorelbine 40 mg three times weekly.

During the final month of her life, she developed frequent episodes of transient dysarthria, paresthesia, as well as vomiting while supine, consistent with clinical progression of LM. Resumption of T-DXd was considered; but her condition deteriorated before treatment could be administered. Following shared decision-making with the patient and her family, the focus of care shifted to hospice care. She died 21 months after the diagnosis of LM. The detailed disease and treatment course are summarized in a timeline (**Figure 4**).

**Figure 4:**
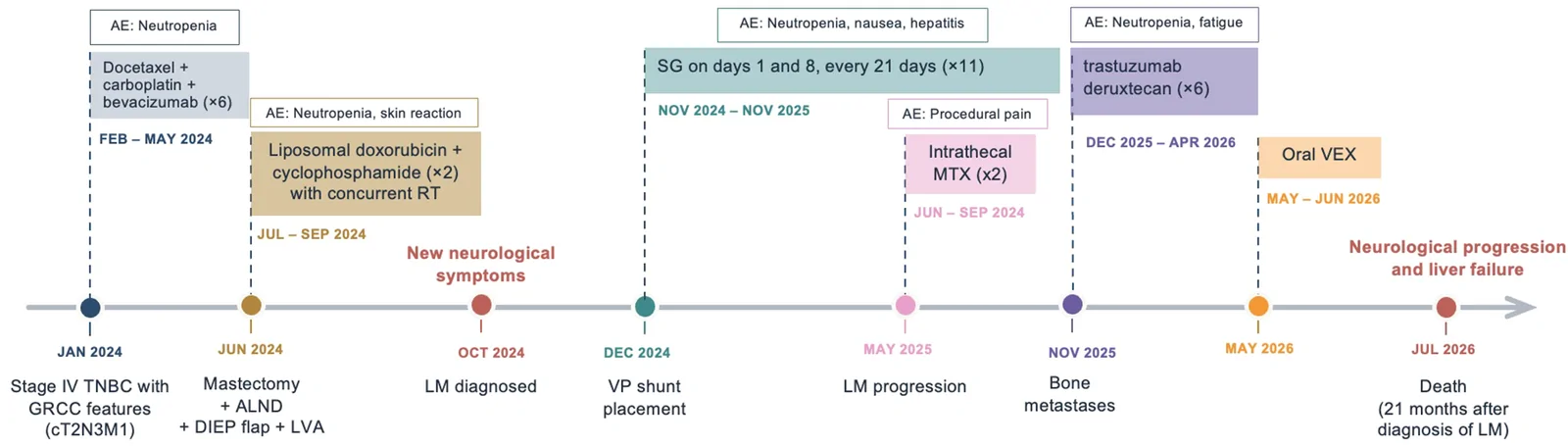
Timeline of the patient’s disease course, treatment, and adverse events. AE, adverse event; TNBC, triple-negative breast cancer; GRCC, glycogen-rich clear cell carcinoma; ALND, axillary lymph node dissection; DIEP, deep inferior epigastric perforator; LVA, lymphovenous anastomosis; RT, radiotherapy; LM, leptomeningeal metastases; VP, ventriculoperitoneal; SG, sacituzumab govitecan; MTX, methotrexate; VEX, vinorelbine, cyclophosphamide, and capecitabine.

## 3 Discussion

Since GRCC carcinoma of the breast was first described in 1981 (5), limited data suggest that this rare subtype may be associated with a poorer prognosis than non-GRCC invasive breast carcinomas, although findings remain inconsistent (6). Current evidence supports management according to molecular subtype and AJCC/TNM staging. To our knowledge, LM arising from breast GRCC have not been previously reported, making this the first documented case.

SG was approved in 2020 for previously treated metastatic TNBC; however, its efficacy in LM remains unclear, as patients with LM are typically excluded from clinical trials. Despite advances in imaging and systemic therapy, outcomes for LM remain poor, with median overall survival of approximately 4–6 months and even shorter survival in TNBC (7). Although treatment is generally noncurative, management of LM is individualized and may integrate disease-directed therapy with supportive care to relieve symptoms, control disease, preserve neurological function, and potentially prolong survival (8).

Only one prior case has reported potential activity of SG in TNBC-associated LM. That patient had a pontine metastasis and extensive craniospinal LM, repeatedly negative CSF cytology, received eight cycles of SG, and died seven months after SG initiation (3). In contrast, our patient had GRCC features, no parenchymal brain metastasis, CSF-confirmed LM, and increased intracranial pressure managed with VP shunt placement. She received 11 cycles of SG plus intrathecal MTX and survived for 21 months after LM diagnosis, maintaining functional independence for most of the follow-up period despite radiographic progression. This longer course may reflect a lower initial neurological burden, relief of intracranial pressure, longer SG exposure, and multimodal therapy; however, it cannot be attributed to SG alone. To our knowledge, this represents the second reported use of SG in TNBC-associated LM and the first in association with GRCC.

Hydrocephalus is a common complication of LM, and VP shunting is an established palliative intervention (9). Although concerns regarding peritoneal dissemination exist, prior studies in metastatic breast cancer have not demonstrated shunt-related peritoneal seeding (10, 11). In our case, no evidence of peritoneal metastasis was observed, raising the hypothesis that VP shunting may influence tumor cell distribution and systemic drug exposure, although this remains speculative.

In conclusion, this case represents the first report of LM arising from GRCC carcinoma of the breast. The prolonged clinical course observed suggests a potential role for SG and VP shunting in LM, warranting further investigation.

## 4 Patient perspective

I did not think too much about the disease itself. When I was diagnosed, I simply followed the treatment plan recommended by my doctors and continued treatment as advised. I did not feel especially positive or negative about it; I just accepted it as part of my daily life.

The headaches and hallucinations occurred when LM was first diagnosed. After the VP shunt was placed, these symptoms improved and did not recur. Except for one seizure episode after becoming overly fatigued, I have been able to tolerate the treatment well. Currently, I am still able to work, travel, and carry out my usual activities. For example, I was able to travel to Turkey for more than 10 days. I hope to continue planning longer trips in the future.

## Data Availability

The original contributions presented in the study are included in the article/supplementary material. Further inquiries can be directed to the corresponding author.
